# Complete genome sequence of *Rhodococcus* sp. strain PD04, a pyridine-degrading bacterium under hypersaline condition

**DOI:** 10.1128/mra.00103-24

**Published:** 2024-06-11

**Authors:** Donghui Huo, Yangfan Hu, Lanpeng Li, Huipeng Gao, Meng Wang, Hao Guan, Quan Zhang, Bo Yu

**Affiliations:** 1 SINOPEC (Dalian) Research Institute of Petroleum and Petro-Chemicals Co., Ltd., Dalian, China; 2 Department of Microbial Physiological & Metabolic Engineering, Institute of Microbiology, Chinese Academy of Sciences, Beijing, China; Indiana University, Bloomington, Indiana, USA

**Keywords:** pyridine degradation, *Rhodococcus *sp., hypersaline

## Abstract

We report the complete genome sequence of a pyridine-degrading *Rhodococcus* sp. strain PD04 under 4% salinity environment, isolated from wastewater of coking plant. The genome is 6.07 Mb with 5,767 annotated gene coding sequences.

## ANNOUNCEMENT

**Fig 1:**
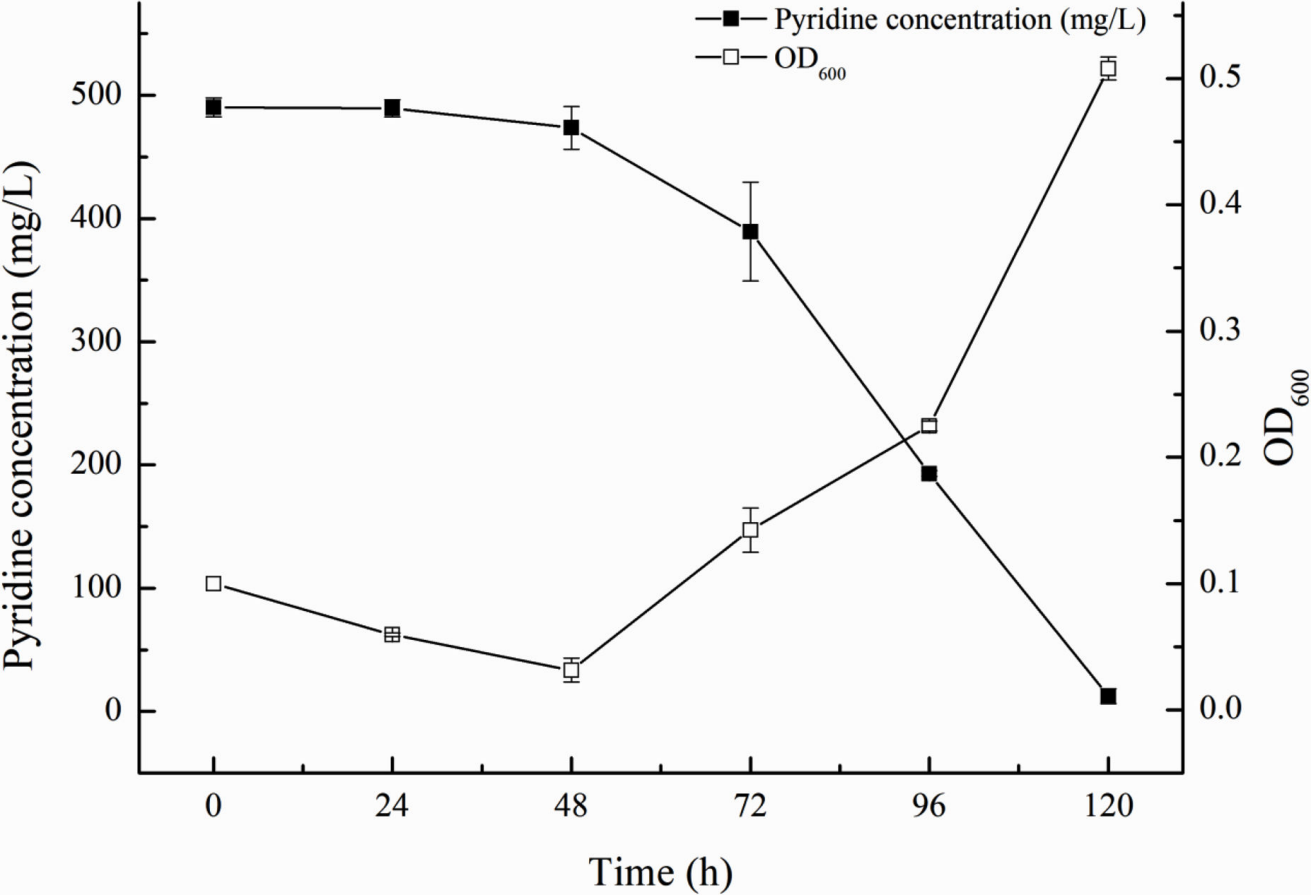
Curves of pyridine degradation and growth of *Rhodococcus* sp. strain PD04 under a salinity of 4% NaCl. The figure shows the mean values of three independent assays. The standard deviation is represented by *error bars*.

Pyridine is an organic pollutant in the wastewater of coal chemical industry, with the characteristic of teratogenicity and carcinogenicity (1, 2). Here, we reported the complete genome sequence of *Rhodococcus* sp. strain PD04, which could grow in the minimal salt medium (3) with 500 mg·L^−1^ pyridine (MSMP) as the sole carbon and nitrogen source under a salinity of 4% NaCl at 30°C (Fig. 1). The density of bacterium and pyridine concentration was determined with multiskan spectrum at 600 nm and 254 nm, respectively (3).

Strain PD04 was isolated from industrial coking wastewater, sampled by fulling 50 mL of sampling bottles from a coking plant in Linyi (118°21' East, 35°06' North), Shandong province, China. The sample was diluted (1:10) into MSMP medium and incubated at 30°C for 4 days for twice, then suspension of 100 µL was directly plated at 30°C on MSMP agar. A single colony was purified three times under the same conditions. Genomic DNA was prepared from an overnight cultured bacterium biomass using the TIANamp Bacteria DNA kit (TIANGEN, China) for the construction of both DNBSEQ and Nanopore libraries, and the genome sequencing was performed at the Beijing Genomics Institution (Shenzhen, China). DNA samples in high quality were sheared randomly by Covaris M220 Focused Acoustic Shearer, and fragments in 300–400-bp were selected by Agencourt AMPure XP-Medium Kit (Beckman Coulter, Inc., USA) for DNBSEQ library construction, and the quality of reads was checked using SOAPnuke v1.5.6 (4). The Nanopore library was prepared without size selection using Ligation Sequencing Kit (SQK-LSK110) and barcoded by Native Barcoding Kit (EXP-NBD104) from Oxford Nanopore Technologies, Britain and sequenced on ONT MinION system with an R9.4.1 flow cell. The ONT reads were base-calling using Guppy v6.5.6 while filtered, keeping reads of >2,000 bp, and checked using porechop v0.2.4 (5). We obtained 8,738,130 raw DNBSEQ reads predicting 213-fold coverage and 212,028 Nanopore long reads with reads N_50_ value of 17,460 bp and an average of 9,114 bp, predicting 318-fold coverage. Subsequently, the filtered reads were corrected and *de novo* assembled with Canu v1.5 using the parameter of estn = 24, npruseGrid = 0 corOvlMemory = 4 (6), and single-base correction was performed with GATK v3.4–0-g7e26428 using the parameter of -cluster 2 -window 5 -stand_call_conf 50 -stand_emit_conf 10.0 -dcov 200 MQ0 >= 4 (7) to obtain a high-confidence assembled sequence.

The assembly generated three contigs, among which the largest was identified as chromosome while others were presumed to be plasmids only based on their size, and was circularized manually by aligning both ends of the contig sequencing and removing overlapping sequences at one end without any rotation. The whole genome comprised one chromosome of 5,307,844 bp, one linear plasmid of 527,914 bp, and one circular plasmid of 237,797 bp, with a total GC content of 67.85%. Gene prediction and annotation were performed by NCBI Prokaryotic Genome Annotation Pipeline (PGAP) with Glimmer v3.02 (8). A total of 5,767 genes in addition to the 54 tRNAs, 12 rRNAs, and 13 sRNAs were annotated. Default parameters were set for all above software unless otherwise specified.

## Data Availability

This whole genome sequence of *Rhodococcus* sp. strain PD04 has been deposited in GenBank under the accession number CP142143-CP142145. The SRA data of DNBSEQ library has been deposited under accession number SRR27586171. The Nanopore library SRA data has been deposited under accession number SRR27586170. This announcement represents the first version of the genome.
